# Predictors of secondary cardiovascular events in older adults with community-acquired pneumonia: a prospective observational study

**DOI:** 10.1016/j.clinsp.2026.100941

**Published:** 2026-04-14

**Authors:** Lianlian Sun, Jialong Zhang, Shi Hu, Rongliang Xu, Pingfa Chen

**Affiliations:** a Internal Medicine, The Second People’s Hospital of Yancheng, Yancheng, Jiangsu, China; b Intensive Care Unit, The Fourth Affiliated Hospital of Nantong University, The First People’s Hospital of Yancheng, Yancheng, Jiangsu, China

**Keywords:** Community-acquired pneumonia, Older adults, Secondary cardiovascular events, Frailty, High-sensitivity troponin

## Abstract

**Background:**

Older adults hospitalized with Community-Acquired Pneumonia (CAP) frequently experience early secondary cardiovascular events. The authors quantified risk factors and the incremental value of frailty and cardiac biomarkers beyond the Pneumonia Severity Index (PSI).

**Methods:**

The authors ran a single-center, prospective cohort of adults ≥65-years with CAP admitted between January and December 2024. The primary endpoint was any in-hospital SCE (acute heart failure, MI/ACS per Fourth Universal Definition, new atrial fibrillation/flutter, ischemic stroke/TIA, or cardiovascular death). Prespecified predictors were prior cardiovascular disease, PSI, Clinical Frailty Scale (CFS), High-Sensitivity Troponin (hs-cTn), and NT-proBNP. Penalized multivariable logistic regression estimated adjusted odds ratios, 30- and 90-day outcomes were used to cause-specific Cox and Fine-Gray models.

**Results:**

Among 172 patients (median age 77; 46% women), in-hospital SCEs occurred in 27.9% patients (48 out of 172 patients with 65 total component events; 95% CI 21.7–35.0), and 68.8% occurred within 72 h. Specifically, heart failure was 18.0%, new atrial fibrillation was 9.9%, MI was 5.8%, and cardiovascular death was 2.9%. In multivariable analysis, CFS ≥ 5 (Aor = 2.07, 95% CI 1.03–4.17), abnormal hs-cTn (aOR = 2.52, 1.25–5.06), and NT-proBNP per doubling (aOR = 1.38, 1.12–1.71) independently predicted SCEs. Discrimination improved from AUC 0.68 (PSI) to 0.73 with CFS and 0.79 with biomarkers (optimism-corrected 0.77), with NRI +0.18 and decision-curve net benefit at 10%–25% thresholds. Cumulative SCE incidence was 33.7% at 30-days and 37.8% at 90-days. Fine-Gray results were consistent.

**Conclusions:**

SCEs were common and occurred early after admission. Adding frailty and cardiac biomarkers to PSI improved risk stratification and may help high-risk patients receive closer monitoring. External validation is needed before its clinical application.

## Highlights

• Secondary Cardiovascular Events (SCEs) occurred in 27.9% of older CAP inpatients.
• Nearly 70% of SCEs developed within 72-hours of hospital admission.
• Clinical Frailty (CFS ≥ 5) independently predicted in-hospital SCEs.
• Adding CFS, hs-cTn and NT-proBNP to PSI raised AUC from 0.68 to 0.79.
• Cumulative SCE incidence reached 33.7% at 30-days and 37.8% at 90-days.

## Introduction

Community-Acquired Pneumonia (CAP) in older adults (≥65-years) remains a leading cause of hospitalization and is frequently complicated by the high incidence of Secondary Cardiovascular Events (SCEs) such as acute heart failure, myocardial infarction, atrial fibrillation, stroke, and cardiovascular death. These events often cluster early during hospitalization and exacerbate patient outcomes.^1^^,^^2^ The occurrence of SCEs is linked to a marked increase in short-term mortality, with studies showing a five-fold increase in 30-day mortality for patients experiencing these complications compared to those who do not.^2^ Risk factors for SCEs include advanced age, high disease severity, and inflammatory markers.^1^^,^^3^ The presence of comorbidities such as hypertension, diabetes, and pre-existing cardiovascular conditions significantly increases the risk of these events.^1^^,^^3^ The burden of CAP in older adults is considerable, with nearly 1 million hospitalizations annually in the United States alone, and over a third of these patients dying within a year.^4^ The high incidence of SCEs and their impact on mortality underscore the need for early identification and management of risk factors to improve outcomes in this vulnerable population.^3^^,^^5^

Bedside tools routinely used in CAP, such as the Pneumonia Severity Index (PSI) and CURB-65, were developed primarily to stratify pneumonia severity and mortality risk rather than cardiovascular complications, and their discrimination for SCEs is therefore expected to be limited.^6^, ^7^, ^8^ Two complementary domains may improve practical SCE risk assessment in older adults with CAP. Frailty (e.g., by the Clinical Frailty Scale, CFS) summarizes physiologic reserve and vulnerability to acute stressors and has been associated with worse outcomes across acute illness, including pneumonia.^9^, ^10^, ^11^ Readily available cardiac biomarkers provide objective signals of myocardial injury and wall stress: high-sensitivity cardiac troponin (hs‑cTn) commonly rises during pneumonia/critical illness and has been associated with short-term cardiovascular complications, while NT‑proBNP reflects cardiac strain and has prognostic value for adverse outcomes in CAP.^12^, ^13^, ^14^, ^15^ Nevertheless, prospective studies in older CAP populations that integrate frailty with cardiac biomarkers and quantify incremental value beyond standard pneumonia severity indices remain limited.^12^^,^^16^^,^^17^

The primary objective is to identify independent risk factors for in-hospital SCEs in older adults (≥65-years) admitted with CAP, using a rigorously defined composite that includes acute heart failure, myocardial infarction/acute coronary syndrome, new-onset atrial fibrillation/flutter, ischemic stroke/transient ischemic attack, and cardiovascular death, adjudicated by blinded clinicians with standardized contemporary criteria. As secondary objectives, the authors will characterize SCE risk through 30- and 90-days from admission, quantify the incremental predictive value of geriatric frailty and readily available cardiac biomarkers beyond established pneumonia severity indices, and delineate the temporal pattern of events with particular attention to early clustering. The study aims to develop a parsimonious, clinically pragmatic risk model that integrates complementary domains, pneumonia severity, frailty (physiologic reserve), and biomarker evidence of myocardial injury and wall stress, to improve bedside risk stratification and guide targeted monitoring in routine care.

## Methods

### Study design

This was a prospective, single-center, non-interventional cohort conducted at The Fourth Affiliated Hospital of Nantong University from January to December 2024, with follow-up through March 2025. Consecutive adults aged ≥65-years admitted to internal medicine or pulmonology wards were screened within 24 h of presentation. The design was observational; no protocol-mandated testing or treatment occurred, and all data were abstracted from routine care supplemented by a brief standardized bedside assessment. The protocol conformed to the Declaration of Helsinki and was approved by the ethics committee of The Fourth Affiliated Hospital of Nantong University (KY2023052). All participants provided written informed consent prior to any study procedures. This study is reported in accordance with the STROBE statement (Supplementary Appendix 1).

### Participants and enrollment

CAP required a new pulmonary infiltrate on chest radiograph or computed tomography plus compatible clinical features (e.g., cough, fever, dyspnea, sputum production, pleuritic chest pain, or focal auscultatory signs). The authors excluded hospital-acquired or ventilator-associated pneumonia, confirmed COVID-19 pneumonia, profound immunosuppression (e.g., post-transplant or chemotherapy-associated neutropenia), and primary cardiovascular admissions where acute myocardial infarction or decompensated heart failure clearly preceded pneumonia.

### Adjudication/blinding

Two clinicians adjudicated cardiovascular endpoints using predefined criteria, and disagreements were resolved by a third adjudicator. Adjudicators were blinded to CFS strata and to any derived risk scores. To adjudicate myocardial infarction per the Fourth Universal Definition, adjudicators had access to clinical charts including ECGs and hs‑cTn results. To mitigate incorporation bias, the authors prespecified a 6‑hour landmark analysis restricting prediction to patients’ event‑free through 6-hours and defined baseline hs‑cTn as the earliest sample obtained prior to any event.

Severe CAP was defined as ≥1 major criterion (septic shock with vasopressors or respiratory failure requiring mechanical ventilation) or ≥ 3 minor criteria (respiratory rate ≥ 30 min; PaO_2_/FiO_2_ ≤ 250; multilobar infiltrates; confusion/disorientation; uremia [BUN ≥ 20 mg/dL]; leukopenia [WBC < 4 × 10^9^/L]; thrombocytopenia [platelets < 100 × 10^9^/L]; hypothermia [< 36 °C]; hypotension requiring aggressive fluid resuscitation).

MI was defined following the Fourth Universal Definition of MI (2018): an acute rise/fall of cardiac troponin with at least one value above the sex‑specific 99th‑percentile URL and evidence of myocardial ischemia (symptoms, new ischemic ECG changes, imaging evidence, or angiographic thrombus). Isolated troponin elevation without ischemia was classified as myocardial injury.

New‑onset Atrial Fibrillation/Flutter (AF) required ECG‑documented AF or typical flutter lasting ≥30 s.

Acute Heart Failure (AHF) (new or decompensated) required typical signs/symptoms (e.g., dyspnea, pulmonary rales, lower‑extremity edema) plus at least one of following criteria: a) Radiographic pulmonary congestion; b) A rise in natriuretic peptides consistent with AHF; or c) Initiation or escalation of IV loop diuretics, vasodilators, or inotropes for congestion/low output, consistent with contemporary HF guidance.

### Predictors and assays

Candidate predictors were prespecified on biological and empirical grounds and limited to reduce overfitting. Prior cardiovascular disease (coronary artery disease, heart failure, or atrial fibrillation), Pneumonia Severity Index (PSI) as the primary severity measure, Clinical Frailty Scale (CFS; 1‒9) assessed by trained staff at admission, high-sensitivity cardiac troponin (hs-cTn I/T), and N-terminal pro-B-type natriuretic peptide (NT-proBNP). PSI entered models as class IV–V vs I–III, and because PSI already incorporates age and sex, these variables were not modeled separately to avoid double-counting. Biomarkers were abstracted from routine care obtained within 24 h of admission; hs-cTn abnormality used the assay-specific, sex-specific 99th-percentile or a clinically meaningful delta when serials were available, and NT-proBNP was modeled on the log2 scale (per doubling). Exploratory variables included the neutrophil-to-lymphocyte ratio, C-reactive protein, and d-dimer when available.

### Sample size and expected events

The target sample was fixed at n = 172 based on site throughput and feasibility. Contemporary elderly CAP cohorts suggest an in-hospital composite SCE incidence of ∼24%–30%, implying 41–52 events. With five prespecified predictors, the authors anticipated ∼8–10 events per parameter and therefore planned penalized regression and bootstrap internal validation to mitigate small-sample bias and overfitting.

### Statistical analysis

Continuous variables were summarized as medians (Interquartile Range, IQR) and categorical variables as counts with percentages. Group differences between patients with and without in-hospital SCE were described with standardized mean differences and compared using Wilcoxon rank-sum tests for continuous variables and χ^2^ or Fisher exact tests for categorical variables. Benjamini-Hochberg False Discovery Rate (FDR) adjustment was applied across baseline comparisons with q-values reported alongside p-values. For proportions, Wilson 95% Confidence Intervals (95% CIs) were used. The primary analysis estimated adjusted associations between prespecified predictors and in-hospital SCE using penalized multivariable logistic regression with Firth bias reduction. Adjusted Odds Ratios (ORs) are presented with profile-likelihood 95% CIs, two-sided p-values, and FDR-adjusted q-values across the five predictors.

*Model development, validation, and comparison*: Because many SCEs occurred very early after admission, the authors prespecified a 6-hour landmark analysis to ensure baseline predictors preceded outcomes and to mitigate potential incorporation bias. The authors developed a parsimonious predictive model for in-hospital SCE with five a priori predictors reflecting chronic substrate (prior cardiovascular disease), acute severity (PSI class IV–V), physiologic reserve (CFS ≥ 5), myocardial injury (abnormal hs-cTn), and myocardial wall stress (NT-proBNP per doubling). Discrimination was quantified by the AUC with 95% CIs and calibration by intercept and slope with a smoothed calibration curve. Overall accuracy was summarized by the Brier score. Internal validation used 1000 resample bootstrap optimism-correction to obtain optimism-adjusted AUC, calibration, and Brier score. To quantify incremental value beyond PSI, the authors compared nested models (PSI only, PSI + CFS, PSI + CFS + biomarkers) using DeLong tests for AUC differences, category-based NRI at prespecified 10% and 20% risk thresholds, IDI, and Decision-Curve Analysis (DCA) to evaluate net benefit across thresholds from 10%–25%.

*Time-to-event analyses and competing risks*: For 30- and 90-day outcomes, time from admission to first SCE was modeled using cause-specific Cox proportional hazards models for etiologic interpretation and Fine-Gray sub-distribution hazards models to estimate cumulative incidence while accounting for the competing risk of non-cardiovascular death. Proportional hazards assumptions were examined using Schoenfeld residuals and log-log plots. The authors present Hazard Ratios (HRs) and Subdistribution HRs (sHRs) with 95% CIs from both frameworks.

*Missing data, sensitivity analyses, and software*: Missing predictors were imputed using multiple imputation by chained equations with ≥ 20 imputations, including all candidate predictors, the outcome indicator, and auxiliary variables. Outcomes were not imputed, and estimates were pooled using Rubin’s rules. Sensitivity analyses included replacing PSI with CURB-65 in the base model, restricting to biomarker-available cases, applying the 6-hour landmark, reclassifying myocardial injury vs. MI with stricter delta criteria, and estimating a cause-specific Cox model for in-hospital SCE, treating discharge as a competing risk. The authors repeated the primary in-hospital SCE model after including the 14 screened patients with confirmed COVID‑19 pneumonia who were excluded from the main CAP cohort, applying the same predictor definitions, imputation strategy, and Firth penalized logistic regression framework. Analyses used R (version 4.4) with packages logistf, pROC, rms, mice, survival, cmprsk/riskRegression, and rmda. All tests were two-sided (α = 0.05).

## Results

**Fig. 1:**
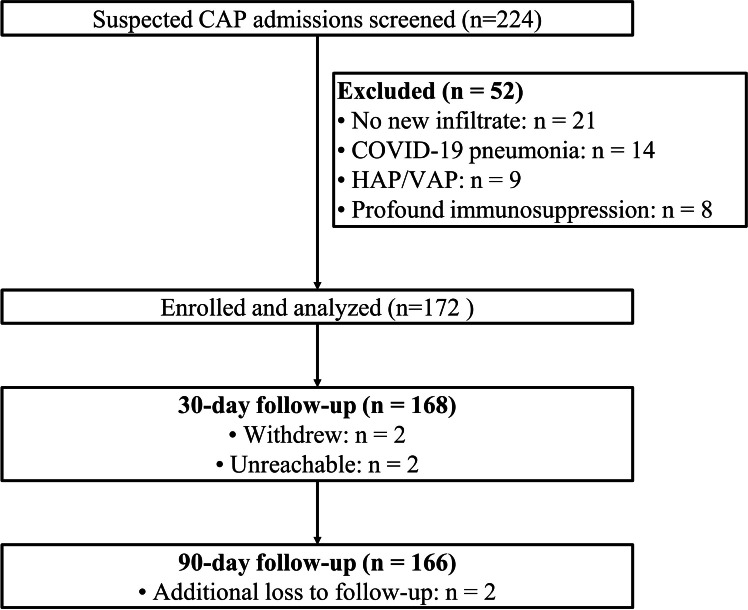
Study flow diagram. Flow of screened patients (n = 224), exclusions with reasons (n = 52), enrolled and analyzed cohort (n = 172), and follow-up completeness at 30- and 90-days.

From January to December in 2024, 224 older adults with suspected CAP were screened, with 52 excluded (21 without a new infiltrate, 14 with confirmed COVID-19 pneumonia, 9 with hospital-acquired/ventilator-associated pneumonia, and 8 with profound immunosuppression), yielding 172 enrolled participants who were all included in the in-hospital analysis (Fig. 1). Follow-up was highly complete, with 168/172 (97.7%) evaluable at 30-days and 166/172 (96.5%) at 90-days (Fig. 1).

The cohort’s median age was 77-years (IQR 71–83), and 45.9% were women. 52.9% of the participants had prior cardiovascular disease, 41.9% presented with PSI class IV–V, 48.8% had CFS ≥ 5, 57.0% had abnormal hs-troponin, and the median NT-proBNP was 1,260 pg/mL (IQR 540–2,380), while 29.1% met ATS/IDSA severe-CAP criteria (Table 1). Compared with those without SCE, patients who developed an in-hospital SCE had higher pneumonia severity (PSI class IV–V 54.2% vs. 37.1%, p = 0.040, FDR q = 0.080), greater frailty (CFS ≥ 5 62.5% vs. 43.5%, p = 0.030, q = 0.070), more frequent abnormal hs-troponin (72.9% vs. 50.8%, p = 0.010, q = 0.030), and higher NT-proBNP (median 1850 vs. 900 pg/mL, p < 0.001, q = 0.002), whereas age showed only a nonsignificant trend and other baseline variables were broadly similar (Table 1).

**Table 1:** Baseline characteristics by in-hospital SCE status.

Characteristic	Overall (n = 172)	SCE (n = 48)	No SCE (n = 124)	SMD	p-value	FDR q
Age (years) median (IQR)	77 (71–83)	79 (73–85)	76 (70–82)	0.28	0.060	0.120
Female, n (%)	79 (45.9%)	21 (43.8%)	58 (46.8%)	0.06	0.730	0.800
Prior cardiovascular disease, n (%)	91 (52.9%)	31 (64.6%)	60 (48.4%)	0.33	0.080	0.140
PSI class IV–V, n (%)	72 (41.9%)	26 (54.2%)	46 (37.1%)	0.35	0.040	0.080
Clinical Frailty Scale ≥5, n (%)	84 (48.8%)	30 (62.5%)	54 (43.5%)	0.39	0.030	0.070
hs-troponin abnormal, n (%)	98 (57.0%)	35 (72.9%)	63 (50.8%)	0.46	0.010	0.030
NT-proBNP, pg/mL, median (IQR)	1260 (540–2380)	1850 (980–3550)	900 (430–1860)	0.51	<0.001	0.002
Neutrophil-to-lymphocyte ratio, median (IQR)	5.0 (3.1–8.0)	5.4 (3.3–8.9)	4.8 (3.0–7.4)	0.17	0.180	0.240
Severe CAP (ATS/IDSA), n (%)	50 (29.1%)	18 (37.5%)	32 (25.8%)	0.25	0.130	0.190
Time to first antibiotic (h) median (IQR)	3.4 (2.1–5.9)	3.2 (2.0–5.3)	3.5 (2.2–6.1)	0.10	0.410	0.480
Oxygen therapy ≥4 L/min or HFNC on admission, n (%)	56 (32.6%)	20 (41.7%)	36 (29.0%)	0.27	0.120	0.180

SCE components were not mutually exclusive. Sixty-five component events occurred among 48 patients with an SCE (48/172; 95% CI 21.7–35.0), with component incidences of acute heart failure 18.0% (31/172), new-onset atrial fibrillation/flutter 9.9% (17/172), myocardial infarction/ACS 5.8% (10/172), stroke/TIA 1.2% (2/172), and cardiovascular death 2.9% (5/172); all-cause in-hospital mortality was 6.4% (11/172) (Table 2). First SCEs clustered early, with 68.8% (33/48; 95% CI 54.7–80.1) occurring within 72-hours of admission. The median time to first SCE was 2.0-days (IQR 1.0–4.0), and the median length of stay was 7-days (IQR 5–10) (Table 2). From admission, cumulative SCE incidence reached 33.7% at 30-days and 37.8% at 90-days (Table 2).

**Table 2:** Cardiovascular event incidence and timing.

Panel A. In-hospital events						
Outcome		Patients with event, n (%)				95% CI (%)
Any SCE (composite)		48 (27.9%)				21.7–35.0
Total SCE events		65 events in 48 patients				
Acute heart failure		31 (18.0%)				13.0–24.4
New-onset atrial fibrillation/flutter		17 (9.9%)				6.3–15.3
Myocardial infarction/ACS		10 (5.8%)				3.2–10.4
Stroke/TIA		2 (1.2%)				0.3–4.1
Cardiovascular death		5 (2.9%)				1.2–6.6
All-cause in-hospital mortality		11 (6.4%)				3.6–11.1

Panel B. Timing of first SCE						
Metric			Value			
Events ≤72 h from admission, n/N (%)			33/48 (68.8%)			
95% CI for ≤72 h proportion (%)			54.7–80.1%			
Median time to first SCE, days (IQR)			2.0 (1.0–4.0)			
Median length of stay, days (IQR)			7 (5–10)			

Panel C. Post-discharge cumulative incidence						
Timepoint	n/N			%	95% CI (%)	
30 days (from admission)	58/172			33.7%	27.1–41.1	
90 days (from admission)	65/172			37.8%	30.9–45.2	

**Fig. 2:**
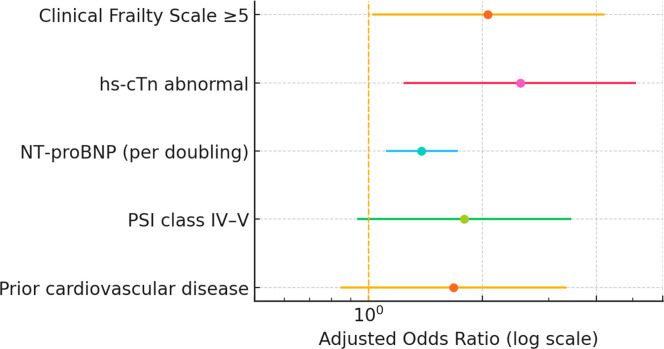
Adjusted odds ratios for in-hospital SCE. Forest plot from the penalized multivariable logistic model showing adjusted odds ratios (ORs, log scale) and 95% CIs for Clinical Frailty Scale ≥5, abnormal hs-troponin, NT-proBNP per doubling, PSI class IV–V, and prior cardiovascular disease.

**Fig. 3:**
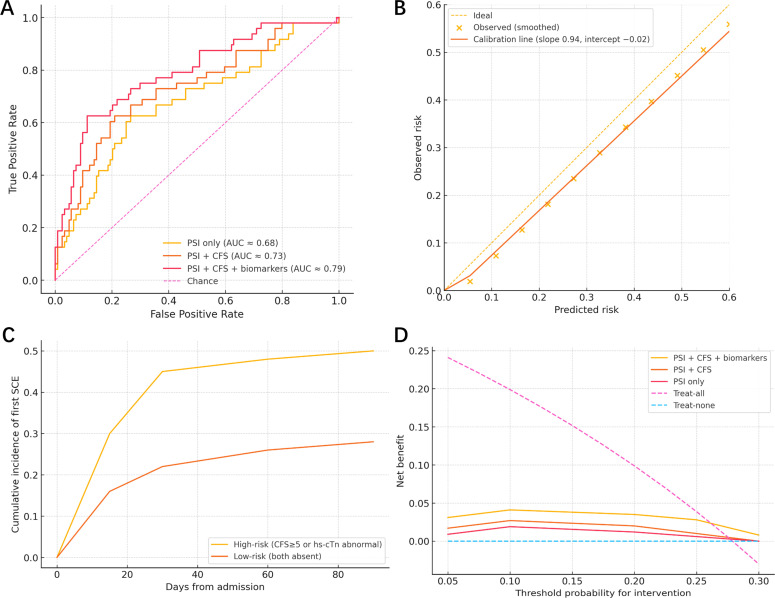
(A) ROC curves for in-hospital SCE models. Discrimination for PSI only (AUC = 0.68), PSI + Clinical Frailty Scale (AUC = 0.73), and PSI + Frailty + biomarkers (AUC = 0.79; optimism-corrected = 0.77); the diagonal indicates chance. (B) Calibration of the final model. Observed vs predicted risks with calibration line (slope 0.94, intercept −0.02) and ideal 45° reference. (C) Fine-Gray cumulative incidence to 90-days. Cumulative incidence of first SCE stratified by a simple high-risk definition (CFS ≥5 or abnormal hs-troponin) vs. low-risk (both absent); curves separate early and remain divergent through 90-days. (D) Decision-curve analysis. Net benefit across threshold probabilities comparing PSI only, PSI + Frailty, and PSI + Frailty + biomarkers, with treat-all and treat-none strategies shown. The biomarker-augmented model yields higher net benefit for thresholds ∼10%–25%.

In the prespecified penalized logistic model including prior cardiovascular disease, PSI class IV–V, CFS ≥ 5, abnormal hs-troponin, and log_2_ NT-proBNP, three predictors were independently associated with in-hospital SCE after adjustment: CFS ≥ 5 (adjusted OR = 2.07, 95% CI 1.03–4.17; p = 0.040; FDR q = 0.066), abnormal hs-troponin (adjusted OR = 2.52, 95% CI 1.25–5.06; p = 0.010; q = 0.025), and NT-proBNP per doubling (adjusted OR = 1.38, 95% CI 1.12–1.71; p = 0.003; q = 0.015). PSI class IV–V showed a borderline association (adjusted OR = 1.79, 95% CI 0.94–3.41; p = 0.080; q = 0.120) and prior cardiovascular disease was not independently significant (adjusted OR = 1.68, 95% CI 0.85–3.31; p = 0.130; q = 0.160) (Table 3 and Fig. 2). The final model demonstrated good discrimination with AUC 0.79 (95% CI 0.72–0.86) and optimism-corrected AUC 0.77, together with sound calibration (slope 0.94, intercept −0.02) and a Brier score of 0.168 (Table 3, Fig. 3A–B). In a sensitivity analysis that included the 14 patients with confirmed COVID‑19 pneumonia (n = 186), the in-hospital SCE incidence was similar to the primary analysis and the multivariable associations were materially unchanged (Supplementary Table S1).

**Table 3:** Multivariable analyses for SCEs.

Panel A. Primary model ‒ In-hospital SCE (penalized logistic regression)									
Predictor (pre-specified)			Adjusted OR		95% CI		p-value	FDR q	
Prior cardiovascular disease (yes vs. no)			1.68		0.85–3.31		0.130	0.160	
PSI class IV–V (vs. I–III)			1.79		0.94–3.41		0.080	0.120	
Clinical Frailty Scale ≥5			2.07		1.03–4.17		0.040	0.066	
hs-troponin abnormal			2.52		1.25–5.06		0.010	0.025	
NT-proBNP (per doubling)			1.38		1.12–1.71		0.003	0.015	

Panel B. Secondary time-to-event ‒ 90-day SCEs									
Predictor	Cause-specific HR	95% CI		p-value		Fine-Gray sHR	95% CI		p-value
Clinical Frailty Scale ≥5	1.66	1.07–2.58		0.024		1.59	1.03–2.45		0.036
hs-troponin abnormal	1.85	1.22–2.81		0.004		1.72	1.13–2.62		0.012
NT-proBNP (per doubling)	1.21	1.04–1.42		0.014		1.18	1.02–1.38		0.026
PSI class IV–V	1.31	0.87–1.98		0.197		1.26	0.83–1.91		0.276
Prior cardiovascular disease	1.22	0.81–1.84		0.343		1.18	0.77–1.80		0.445

Over 90-days, both cause-specific and sub-distribution analyses confirmed that frailty and cardiac biomarkers predicted first SCEs. In Fine-Gray models treating non-cardiovascular death as a competing event, CFS ≥ 5 (sHR = 1.59, 95% CI 1.03–2.45; p = 0.036), abnormal hs-troponin (sHR = 1.72, 95% CI 1.13–2.62; p = 0.012), and NT-proBNP per doubling (sHR = 1.18, 95% CI 1.02–1.38; p = 0.026) remained significant, whereas PSI class IV–V and prior cardiovascular disease were not independently associated. Cause-specific Cox estimates were directionally similar (Table 3, Fig. 3C).

Adding frailty and biomarkers to a PSI-only model yielded incremental improvements in discrimination, reclassification, and net benefit. The AUC increased from 0.68 (95% CI 0.60–0.76) with PSI alone to 0.73 (95% CI 0.66–0.81) with PSI + CFS (DeLong p = 0.040), and to 0.79 (95% CI 0.72–0.86) with PSI + CFS + biomarkers (DeLong p = 0.010), with optimism-corrected AUCs of 0.66, 0.71, and 0.77, respectively. Calibration remained acceptable, and overall accuracy improved as the Brier score decreased from 0.200 to 0.168 (Table 4, Fig. 3A–B). Relative to PSI alone, category-based NRI was +0.11 (95% CI 0.00–0.23; p = 0.050) after adding CFS and +0.18 (95% CI 0.05–0.31; p = 0.006) after adding biomarkers, with parallel gains in IDI (+0.020, 95% CI 0.002–0.038; p = 0.029; and +0.040, 95% CI 0.010–0.070; p = 0.010), and decision-curve analysis showed higher net benefit for the biomarker-augmented model across 10%–25% risk thresholds (Table 4, Fig. 3D).

**Table 4:** Model comparison: incremental value beyond PSI.

Metric	PSI only	PSI + CFS	PSI + CFS + biomarkers (hs-cTn, NT-proBNP)
AUC (95% CI)	0.68 (0.60–0.76)	0.73 (0.66–0.81)	0.79 (0.72–0.86)
Optimism-corrected AUC	0.66	0.71	0.77
DeLong p vs. previous model	–	0.040	0.010
Calibration slope/intercept	0.98 / −0.01	0.96 / −0.01	0.94 / −0.02
Brier score	0.200	0.186	0.168
NRI vs. PSI (95% CI), p	–	+0.11 (0.00–0.23), 0.050	+0.18 (0.05–0.31), 0.006
IDI vs. PSI (95% CI), p	–	+0.020 (0.002–0.038), 0.029	+0.040 (0.010–0.070), 0.010
Net benefit at 10% risk	0.019	0.027	0.041
Net benefit at 20% risk	0.012	0.020	0.035

## Discussion

In this prospective single-center cohort of older adults with CAP, SCEs were common and occurred early, driven primarily by acute heart failure, with additional contributions from new-onset atrial fibrillation, myocardial infarction, and cardiovascular death. In the prespecified penalized multivariable model that adjusted for PSI, frailty, myocardial injury, and myocardial wall stress independently identified patients at risk, while exploratory inflammatory markers, including NLR, showed only modest unadjusted associations and were not independently associated after adjustment. Beyond PSI alone, adding CFS increased discrimination, and adding hs-cTn and NT-proBNP further increased the discrimination capacity.

In older adults with CAP, this data show a high early burden of SCEs with 27.9% in-hospital and 68.8% within 72 h, which is consistent with a “susceptibility + trigger” paradigm in which chronic substrate and acute stressors intersect. The NLR reflects infection-driven inflammatory imbalance and immunothrombosis, mechanistically linked to endothelial dysfunction, plaque instability, and proarrhythmia. Prior syntheses associate higher NLR with adverse outcomes in CAP and with AF risk.^18^, ^19^, ^20^, ^21^ Hs-cTn elevation during CAP indicates myocardial injury from supply-demand mismatch, microvascular ischemia, or direct inflammatory toxicity, and predicts near-term cardiovascular complications when distinguished from MI per the Fourth Universal Definition, mirroring the independent association for abnormal hs-cTn.^12^^,^^13^^,^^22^ NT-proBNP, indexing myocardial wall stress and congestion, was likewise independently associated with SCEs in our cohort and has shown prognostic value for death and heart-failure-type events in CAP.^14^^,^^15^ Superimposed frailty captures diminished physiologic reserve and dysregulated stress responses, amplifying vulnerability to the same triggers, thus integrating with biomarkers to operationalize the shared pathway to acute heart failure, AF, MI, and ischemic stroke/TIA in CAP.^2^^,^^10^^,^^11^^,^^17^

The in-hospital SCE rate and early clustering align with prospective cohorts of hospitalized CAP, particularly the multicenter ICECAP study and earlier work by Violi et al., and exceed pooled estimates from mixed-age or retrospective series in which outcome definitions varied and myocardial injury was often conflated with MI.^2^^,^^17^^,^^23^ By using blinded adjudication under the Fourth Universal Definition of MI, the authors separated injury from type 1/type 2 MI, improving internal validity and comparability.^22^ Beyond reproducing known signals for pneumonia severity, the authors demonstrate incremental prediction from hs-cTn and NT-proBNP and add a geriatric dimension (frailty) often absent from prior models, with stepwise improvements in discrimination and clinical utility.^12^^,^^14^^,^^15^

At admission for CAP, a pragmatic enrichment strategy, CFS plus hs-cTn and NT-proBNP, can flag older adults for earlier telemetry, heart-failure-oriented assessment (including judicious natriuretic peptide use), and closer hemodynamic/oxygenation monitoring, complementing ATS/IDSA severity guidance.^6^^,^^12^^,^^19^^,^^24^^,^^25^ At discharge and early follow-up, the persistently elevated 30–90 day cardiovascular risk after pneumonia justifies targeted surveillance for arrhythmias and heart-failure decompensation, medication reconciliation, and risk-factor management.^26^ Decision-curve results in the present study’s cohort suggest that models incorporating frailty and cardiac biomarkers yield net clinical benefit across commonly considered thresholds, supporting risk-guided monitoring pathways rather than one-size-fits-all protocols.^27^

This study has several important limitations that should temper interpretation. First, confirmed COVID‑19 pneumonia was excluded, which narrows generalizability to contemporary practice where viral pneumonias, including SARS‑CoV‑2, are common. Although this exclusion was intended to preserve etiologic and management homogeneity, it may introduce selection bias and limit inference to non‑COVID CAP populations; validation in cohorts that include viral pneumonia is needed. Second, the sample size and event count were modest, yielding a relatively low events-per-parameter ratio for multivariable modeling and increasing the risk of overfitting and unstable effect estimates. The authors attempted to mitigate this by prespecifying a small set of clinically motivated predictors, using Firth penalization, and performing bootstrap optimism correction. Nonetheless, residual optimism and model instability remain possible. Third, the model lacks external validation. All data were derived from a single center with local practice patterns. Independent external multicenter validation will be necessary before any consideration of clinical implementation. Fourth, the clinical utility of admission-time risk stratification is constrained by the very early timing of events in the present cohort, with most first SCEs occurring within the first 72-hours. Even with a landmark approach to reduce incorporation bias, some predictors may reflect evolving physiology close to the event window, limiting the extent to which the model can be interpreted as enabling prevention rather than contemporaneous risk identification. Therefore, our findings support risk enrichment for early monitoring and follow-up planning, but do not demonstrate that biomarker- or frailty-guided interventions prevent SCEs or improve outcomes. Additional limitations include the observational design with potential residual confounding, and reliance on routine-care biomarker testing (which may be subject to selection effects despite imputation and sensitivity analyses). Future work should focus on multicenter validation and prospective evaluation of risk-guided monitoring or care pathways.

In this prospective cohort of older adults hospitalized with CAP, a parsimonious, practice‑ready approach that integrates pneumonia severity, geriatric frailty, and cardiac biomarkers, with NLR as an adjunct inflammatory signal, substantially improved early cardiovascular risk stratification beyond severity alone. These findings support further multicenter validation and prospective evaluation of whether risk-enriched monitoring strategies can improve outcomes.

## Ethics approval and consent to participate

The protocol conformed to the Declaration of Helsinki and was approved by the ethics committee of The Fourth Affiliated Hospital of Nantong University (KY2023052). All participants provided written informed consent prior to any study procedures.

## Data availability

De-identified individual participant data, related analysis, and results generated during the current study are available from the corresponding author on reasonable request.

## Clinical trial number

Not applicable.

## Consent to publish declaration

Not applicable.

## Authors’ contributions

The authors confirm contribution to the paper as follows: study conception and design: P.C.; data collection: L.S., J.Z., S.H., R.X.; analysis and interpretation of results: L.S., J.Z., S.H., R.X.; draft manuscript preparation: L.S., J.Z., S.H., R.X., P.C. All authors reviewed the results and approved the final version of the manuscript.

## Funding

This study received no external funding. Biomarker testing was performed as part of routine clinical care, and study coordination and follow-up were supported by departmental resources.

## Conflicts of interest

The authors declare no conflicts of interest.

## Appendix Supplementary materials

Image, application 1

## Footnotes

Supplementary material associated with this article can be found, in the online version, at doi:10.1016/j.clinsp.2026.100941.
